# Identifying fatigue of climbing workers using physiological data based on the XGBoost algorithm

**DOI:** 10.3389/fpubh.2024.1462675

**Published:** 2024-10-09

**Authors:** Yonggang Xu, Qingzhi Jian, Kunshuang Zhu, Mingjun Wang, Wei Hou, Zichao Gong, Mingkai Xu, Kai Cui

**Affiliations:** ^1^Emergency Management Center of State Grid Shandong Electric Power Company, Jinan, China; ^2^State Grid Shandong Electric Power Company, Jinan, China; ^3^School of Modern Postal, Xi'an University of Posts and Telecommunications, Xi'an, China

**Keywords:** fatigue identification, climbing workers, physiological data, machine learning, XGBoost

## Abstract

**Background:**

High-voltage workers often experience fatigue due to the physically demanding nature of climbing in dynamic and complex environments, which negatively impacts their motor and mental abilities. Effective monitoring is necessary to ensure safety.

**Methods:**

This study proposed an experimental method to quantify fatigue in climbing operations. We collected subjective fatigue (using the RPE scale) and objective fatigue data, including systolic blood pressure (SBP), diastolic blood pressure (DBP), blood oxygen saturation (SpO_2_), vital capacity (VC), grip strength (GS), response time (RT), critical fusion frequency (CFF), and heart rate (HR) from 33 high-voltage workers before and after climbing tasks. The XGBoost algorithm was applied to establish a fatigue identification model.

**Results:**

The analysis showed that the physiological indicators of SpO_2_, VC, GS, RT, and CFF can effectively evaluate fatigue in climbing operations. The XGBoost fatigue identification model, based on subjective fatigue and the five physiological indicators, achieved an average accuracy of 89.75%.

**Conclusion:**

This study provides a basis for personalized management of fatigue in climbing operations, enabling timely detection of their fatigue states and implementation of corresponding measures to minimize the likelihood of accidents.

## 1 Introduction

In recent years, the escalating societal demand for electricity has precipitated a surge in power equipment failures, underscoring the pressing need for a stable power supply and seamless operational continuity. Essential to this mandate are climbing operations, routine maintenance, and the prompt rectification of latent faults within the power grid, all pivotal for upholding its unimpeded functionality (1). Regrettably, incidents involving safety breaches among climbing operations have become commonplace during these operations. These occurrences not only imperil workers' safety and wellbeing but also exact a profound toll on the psychological and financial fabric of families and enterprises. Moreover, they impede the overarching economic advancement of the industry. According to incomplete records from the National Energy Administration-Electric Power Safety Supervision Department, in 2022, China witnessed 24 power-related personal injury accidents, resulting in 34 fatalities (2). Notably, 10 of these incidents involved falls from considerable heights, constituting 42% of the total accidents, with 12 deaths stemming from these falls, accounting for 35.3% of the overall fatalities. A meticulous analysis of these accidents elucidates that the unsafe conduct of personnel engaged in climbing operations serves as the principal catalyst for falls from elevated positions, with fatigue emerging as the primary internal determinant precipitating these hazardous behaviors (3, 4). Fatigue manifests through compromised productivity, diminished focus, delayed cognitive response, languid motor function, and sundry unsafe behaviors (5). Hence, a comprehensive exploration of fatigue dynamics during climbing operations assumes paramount importance in forestalling or mitigating incidents of falls from heights.

In the past, traditional research methods primarily relied on surveys and interviews to collect self-perceived feelings and evaluations of workers, which are indicative of subjective fatigue (6, 7). However, although these methods are simple and easy to implement, they often need to be more objective and accurately reflect the actual fatigue states of workers. During prolonged physical activities, especially in high-intensity labor like climbing operations, individuals often experience fatigue. At this time, significant changes occur in their various physiological indicators. These objective alterations, including reaction times and metabolic rates, can directly reflect objective fatigue states (7, 8). Liang et al. conducted in-depth analysis and research on the localized muscle fatigue of climbing workers using advanced techniques such as electromyography (EEG). They found a close correlation between the characteristic changes in surface electromyography signals during operations and subjective fatigue assessment values (9). Ma et al. proposed a new posture prediction and analysis method by comparing and analyzing the differences in posture between workers under fatigue and non-fatigue conditions. They found that workers' posture under fatigue conditions often differs from that under normal conditions, providing essential clues for evaluating workers' physiological fatigue. This research offers new insights and methods for assessing the fatigue of climbing workers (10). Zhou et al. collected four physiological indicators of climbing workers. They combined them with subjective fatigue level scales to comprehensively assess the fatigue states of workers. They used support vector machines to build fatigue detection models and experimentally verified the feasibility and effectiveness of this method (11).

Combining objective and subjective methods is considered accurate and practical for assessing the fatigue states (12). This approach finds the workers' physiological responses and subjective feelings. This comprehensive and detailed assessment method can more accurately reflect the fatigue states of climbing workers, thus supporting their work safety and health. However, current research primarily focuses on laboratory environments, simulating climbing operations to investigate worker fatigue state changes (13). Although this research method is convenient, it can only partially replicate on-site climbing operations' natural environment and complexity. Therefore, more research is needed on the fatigue states of climbing workers in actual field conditions. Power operation sites constitute a complex and dynamically changing human-machine-environment-management system (14, 15). In this system, workers must face various unpredictable factors such as weather changes, equipment failures, and changes in job tasks. These factors may affect the fatigue states, and laboratory environments are challenging to simulate completely in these real situations.

Consequently, there often needs to be more existing experimental data and on-site operations. To better understand and assess the fatigue states of climbing workers, we need to focus more on collecting data from real work sites. By gathering physiological data, psychological perceptions, and job environment information from on-site workers, we can comprehensively understand their fatigue state changes, providing more targeted recommendations for improving their working conditions and enhancing work safety. Moreover, this will also offer more prosperous and more authentic data support for our future research endeavors.

Considering the above factors, the present article advances a methodological framework for fatigue measurement in climbing operations, incorporating subjective and objective data. The quantification of fatigue states is achieved through the comprehensive collection of subjective fatigue scales, reflecting the workers' self-assessed experiences and diverse physiological indicators, providing an objective measurement of their fatigue states. Furthermore, implementing the XGBoost algorithm is pivotal in crafting a fatigue prediction model explicitly tailored for climbing operations. This approach bears profound practical significance as it facilitates real-time fatigue monitoring and issuance of timely fatigue warnings for climbing operation personnel. The amalgamation of subjective and objective data, coupled with advanced predictive modeling techniques, not only enhances the precision of fatigue assessment but also underscores the pragmatic utility of the proposed methodology in safeguarding the wellbeing and operational efficiency of power climbing operation personnel.

The significance of this study is as follows:

(1) Contribution to theory: this research enriches the theoretical framework of fatigue measurement by integrating subjective and objective aspects into a cohesive model, providing a more comprehensive understanding of fatigue in physically demanding tasks.
(2) Methodological advancement: by employing a combination of subjective fatigue scales and objective physiological indicators alongside the XGBoost algorithm, the study offers a robust and multifaceted methodology for assessing fatigue in climbing operations.
(3) Empirical data utilization: applying this methodology to empirical data from climbing operations allows the study to provide grounded and context-specific insights, enhancing the field's ability to monitor and mitigate fatigue-related risks in real-world scenarios.

## 2 Material and methods

### 2.1 Selection of subjects

Stringent criteria were implemented for participant selection to mitigate interference from inter-participant physical variations in measuring physiological indicators. Specifically, participants were required to fall within a specified Body Mass Index (BMI) range and exhibit good physical health without any history of illness (16). Following a rigorous screening process, a cohort of 33 male participants, aged between 26 and 32 years old, was selected from a power supply company's training center to participate in the experiment. Participants were instructed to adhere to specific guidelines to control for external factors that could impact the experiment. These guidelines included abstaining from staying up late, refraining from alcohol consumption, and avoiding the use of medication (17). Before the commencement of the experiment, participants were obligated to provide informed consent by signing a consent form. Furthermore, participants underwent comprehensive briefings and explanations to ensure a thorough understanding and cooperation throughout the experimental procedures. This study has received ethical approval from the relevant ethics committee, and the entire experimental process strictly adheres to established safety protocols.

### 2.2 Experimental indicators and data collection

In consonance with the operational characteristics inherent to power pole activities, the assessment of subjects' fatigue levels encompasses the examination of eight selected physiological indicators, including systolic blood pressure (SBP), diastolic blood pressure (DBP), blood oxygen saturation (SpO_2_), vital capacity (VC), grip strength (GS), response time (RT), critical fusion frequency (CFF) and heart rate (HR). The selected physiological indicators are designed to comprehensively cover various aspects of the cardiovascular, respiratory, and muscular systems, providing a multidimensional perspective on fatigue monitoring. In the existing literature, these indicators have been widely used to evaluate fatigue across various groups, including athletes, drivers, and healthcare workers. The fluctuations in data before and after fatigue are well-documented and widely accepted (Table 1). Additionally, compared to technologies such as eye-tracking and EEG, these physiological indicators are more convenient to measure, cost-effective, and straightforward to process, with results that are easy to interpret. This makes them particularly advantageous for practical fatigue monitoring applications.

**Table 1:** Previous studies on measurement indicators.

**Constructs**	**Indicators**	**Sources**
Subjective fatigue	RPE	Zhou et al. (11), Lea et al. (18), Bok et al. (19)
Objective fatigue	SBP	Richard et al. (20), Mun and Geng (21)
	DBP	Mun and Geng (21), Guest et al. (22)
	SpO_2_	Jagannath and Balasubramanian (23), Putra et al. (24)
	VC	Chen et al. (25), Taylor et al. (26)
	GS	Starling-Smith et al. (27), Xu (28)
	RT	Chen et al. (25), Migliaccio et al. (29)
	CFF	Song et al. (30), Łuczak and Sobolewski (31)
	HR	Putra et al. (24), Chen et al. (25)

The experimental data collection process entailed the deployment of six distinct instruments. Participants were instructed to sanitize their left index finger with an alcohol swab and affix the YX306 fingertip heart rate and blood oxygen saturation. SpO_2_ and HR readings were meticulously recorded after device activation and the requisite stabilization period. Subjects were instructed to don the U10L blood pressure cuff on their left upper arm, with the lower edge ~2 centimeters above the elbow pit, to acquire SBP and DBP data. VC measurements were executed using the FCS-10000 spirometer. The BD-II-118 flash frequency meter facilitated the assessment of CFF, involving three consecutive measurements, with the resultant average value adopted. GS data were garnered through the CAMRY grip strength meter, with subjects instructed to exert maximal force during a gripping maneuver lasting no longer than 2 s. RT data were procured through a visual choice task programmed on the E-prime software platform, demanding prompt and accurate responses to stimuli of four distinct colors: red, green, blue, and yellow. Additionally, the Rate of Perceived Exertion (RPE) scale was employed as the chosen subjective indicator to quantify subjects' perceived fatigue levels.

### 2.3 Experimental implementation

The experiments were conducted within the confines of the Climbing Training Center's office space, where environmental conditions such as temperature, humidity, and lighting were deliberately regulated to moderate levels. These experiments were scheduled in early July, encompassing morning and afternoon sessions, precisely at 8 a.m., 12 p.m., 2 p.m., and 6 p.m. These time slots were strategically chosen to correspond with the pre and post-training sessions for the participants undergoing extensive power system maintenance training during this designated period at the center. The selection of July was intentional, aligning with the center's training schedule and ensuring the participants' consistent engagement in rigorous daily power system maintenance training. Furthermore, the training center imposed standardized routines on the participants, ensuring uniformity in their daily activities and living conditions. This collective approach rendered this period both representative and significant for the study.

**Figure 1:**
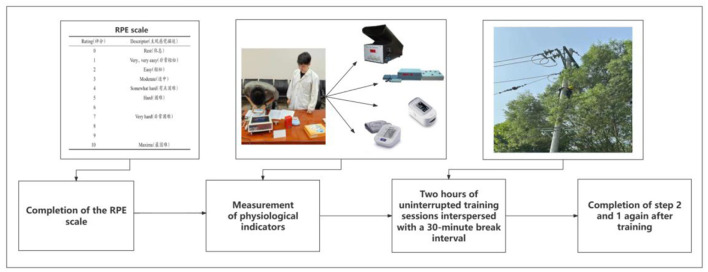
Experimental flow chart.

In order to mitigate individual variability within a single day of measurement, each participant was required to partake in a minimum of 3 days of repeated measurements, resulting in at least 12 sets of data per individual. Before the commencement of the experiment, all 33 participants were assigned identification numbers and provided with comprehensive instructions on utilizing the measuring instruments. Moreover, the parameters of each instrument were meticulously set in advance to ensure consistency and uniformity in the measurement procedures across all participants. The flow of the experiment is shown in Figure 1. The formal experimental protocol was as follows:

(1) Test participants' subjective fatigue levels using the RPE scale before training.
(2) The sequential performance of measurements using the six designated instruments, encompassing the entire process from start to finish, takes ~7 min to complete. During this period, experimental data are systematically recorded. Following the measurements, participants undergo two uninterrupted training sessions, each separated by a 30-min break interval.
(3) After completing the training regimen, participants provided updated ratings on the RPE scale based on their current condition, and post-fatigue data was diligently collected for analysis.

## 3 Data analysis

For the data samples collected in the experiment, 435 sets of valid data were obtained after cleaning missing values and outliers. Descriptive statistics (mean and standard deviation) were performed on all indicators in this data sample to understand the essential characteristics of the data comprehensively.

### 3.1 RPE scale data

The Rating of Perceived Exertion (RPE) scale is widely used to assess subjective perceptions of effort during physical activity. Initially ranging from 6 to 20, the scale has been adapted to various formats, including the Borg CR-10 scale, which ranges from 0 to 10, with 0 indicating no exertion and 10 indicating maximal exertion. While initially devised for use during exercise, the RPE scale has been extended to measure fatigue in other domains, such as occupational settings and clinical research. Participants are asked to rate their perceived exertion or fatigue level based on their subjective interpretation of effort, providing valuable insights into their perceived levels of fatigue and exertion during tasks (32, 33). The RPE scale offers a simple yet effective means of quantifying subjective feelings of fatigue, making it a valuable tool for assessing fatigue across different contexts and populations. In line with the actual situation of workers, this study utilized the RPE scale to gather subjective fatigue levels, where one indicates very relaxed, and ten indicates highly exhausted (11).

The frequency analysis results of the RPE scale data are presented in Table 2. Participant-perceived fatigue levels predominantly fall within the range of levels 3 to 6. Specifically, data samples indicating a fatigue level of 5 exhibit the highest frequency, totaling 85 samples and representing 19.54% of the overall dataset. Following closely, fatigue level 4 demonstrates the second-highest frequency, with 82 samples constituting 18.85% of the total. Conversely, the least frequent perceived fatigue level is level 9, comprising seven samples and accounting for 1.61% of the entire dataset. The second lowest frequency is observed at level 10, with nine samples constituting 2.07% of the total. To facilitate more accurate data analysis subsequently, researchers typically divide fatigue into three levels based on the characteristics of the RPE scale (34, 35). Specifically, fatigue levels 1 and 2 were defined as no fatigued state, indicating participants feeling relaxed or slightly fatigued. Subsequently, samples with fatigue levels 3, 4, 5, and 6 were defined as mild fatigue state, reflecting participants beginning to feel a certain degree of fatigue while still maintaining typical work efficiency. Finally, samples with fatigue levels 7, 8, 9, and 10 were defined as extreme fatigue state, indicating participants experiencing severe fatigue during electrical pole climbing tasks, which may adversely affect their work efficiency and safety.

**Table 2:** The frequency analysis results of the RPE scale.

**Rating**	**1**	**2**	**3**	**4**	**5**	**6**	**7**	**8**	**9**	**10**
Frequency	45	31	55	82	85	57	35	29	7	9

Furthermore, a chi-square test was employed to assess the potential significant differences between participants and the RPE scale data. The results indicate a lack of statistically significant differences between participants and the RPE scale data (*P* = 0.855), suggesting that perceived fatigue levels were comparable among different participants.

### 3.2 Differences in physiological indicators of fatigue

The data are typically distributed, allowing for further analysis of various physiological indicators over time (as shown in Table 3) for variables such as SBP, GS, RT, SpO_2_, VC, and CFF, which meet the assumption of variance homogeneity, a one-way variance analysis (ANOVA) was conducted with a significance level set at *P* = 0.05. The resulting *P*-values were 0.000, 0.014, 0.085, 0.309, 0.498, and 0.203, respectively, indicating significant differences in SBP and GS over time. Welch's ANOVA was utilized for HR and DBP, which did not meet the homogeneity of variance assumption. Both showed *P*-values of 0.000, signifying significant temporal variations in HR and DBP. Specifically, SBP, DBP, GS, and HR show significant daily fluctuations. SBP remains relatively consistent in the morning, noon, and afternoon but rises to 124.12 with a standard deviation of 8.8 by 6 p.m. DBP is notably lower at 8 a.m. compared to other times. GS averages 47.07 at 8 a.m. with a standard deviation of 8.52, decreases to 45.62 and 49.13 at noon and 2 p.m., with standard deviations of 8.15 and 8.66 respectively, and slightly decreases to 47.44 with a standard deviation of 8.85 by 6 p.m. HR shows some variation throughout the day. VC, GS, RT, and HR standard deviations are relatively large, indicating significant differences in grip strength among different individuals.

**Table 3:** Physiological indicator data at different time.

**Indicators**	**Descriptive statistics**	**Time**			
		**8 a.m**.	**12 a.m**.	**2 p.m**.	**6 p.m**.
SBP	*M*	119.25	116.65	118.8	124.12
	SD	8.93	9.76	10.68	8.8
DBP	*M*	73.09	71.49	73.8	77.26
	SD	6.82	7.13	8.51	7.93
SpO_2_	*M*	97.48	97.53	97.59	97.79
	SD	1.1	1.06	1.15	0.87
VC	*M*	4,267.54	4,259.93	4,380.44	4,318.57
	SD	711.34	758.97	658.93	750.54
GS	*M*	47.07	45.62	49.13	47.44
	SD	8.52	8.15	8.66	8.85
RT	*M*	554.53	561.84	526.64	553.13
	SD	121.23	122.88	111.01	113.9
CFF	*M*	35.93	35.5	36.18	35.73
	SD	2.35	2.36	2.63	2.97
HR	*M*	82.23	77.36	83.78	77.55
	SD	9.7	10.33	14.82	10.36

#### 3.2.1 SBP

**Figure 2:**
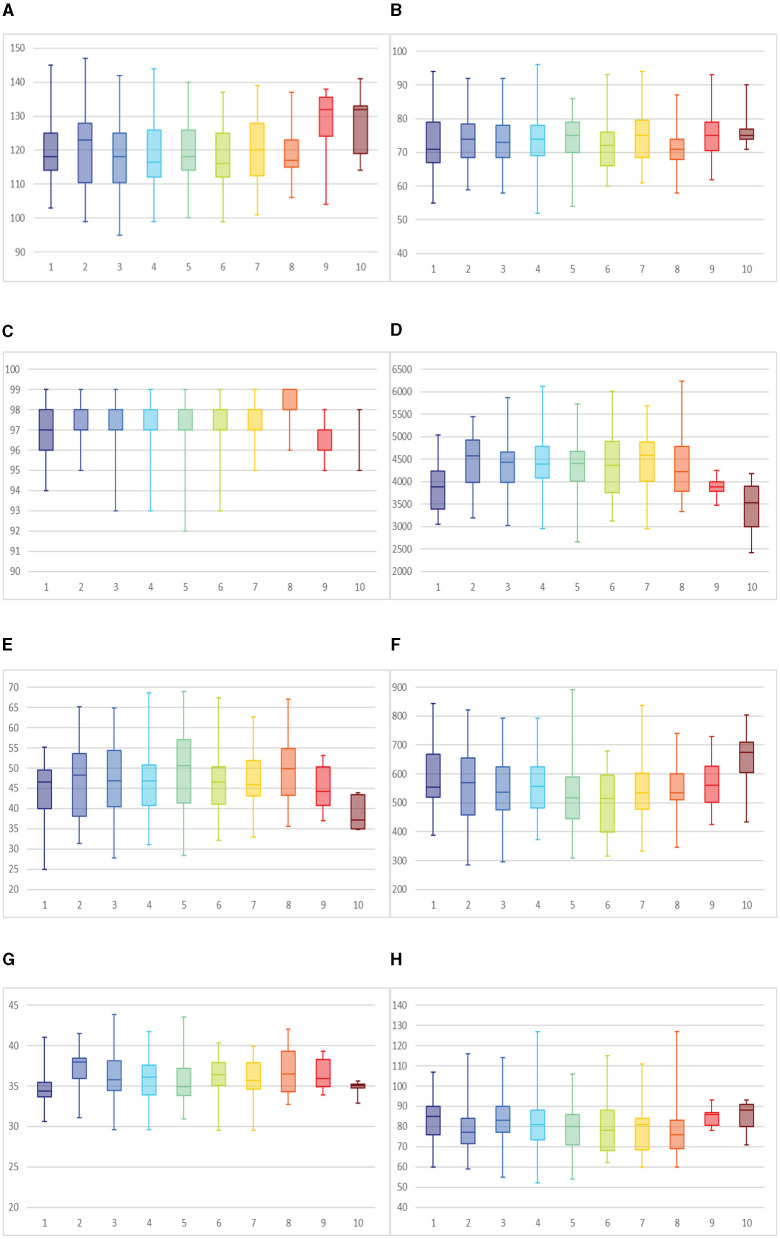
Physiological indicator data at different fatigue levels: **(A)** SBP, **(B)** DBP, **(C)** SpO_2_, **(D)** VC, **(E)** GS, **(F)** RT, **(G)** CCF, and **(H)** HR.

SBP refers to the lateral pressure exerted by the blood flow against the vessel wall during cardiac contraction, reflecting one of the physiological indicators of average circulation and serving as a crucial vital sign. Numerous experiments have shown that intense or prolonged physical activity can lead to a rapid and temporary increase in blood pressure, potentially increasing the risk of heart disease, stroke, or other cardiovascular diseases in hypertensive individuals, thereby endangering their lives (36, 37). For adult males, the typical range for average SBP is usually between 90–140 mmHg (38). Figure 2A depicts that some electric pole-climbing workers have SBP readings ranging from 140–150 mmHg, exceeding the normal range. Further analysis of SBP differences under varying fatigue levels reveals that SBP is higher at 9 (*M* = 127.57, SD = 12.11) and 10 (*M* = 127.67, SD = 9.82) than other fatigue states. This suggests that as fatigue deepens, there is an increasing trend in SBP among workers. However, this difference is not statistically significant (*P* = 0.091 > 0.05).

#### 3.2.2 DBP

DBP refers to the pressure in the arteries during heart relaxation, which is crucial for evaluating the blood circulation status and overall health of the body. Adult males' normal diastolic blood pressure typically ranges from 60–90 mmHg (38). Figure 2B shows that some individuals have high DBP, reaching 90–100 mmHg, while others have low DBP, ranging between 50–60 mmHg. Multiple-group data one-way ANOVA analysis indicates no significant difference in DBP among different fatigue levels (*P* = 0.662 > 0.05), suggesting no apparent trend in diastolic pressure levels among individuals.

#### 3.2.3 SpO_2_

SpO_2_ refers to the percentage of oxygen content in the blood relative to its maximum capacity. Normal SpO_2_ is typically above 95%. Generally, the onset of fatigue leads to a decrease in SpO_2_ among healthy individuals (39). This occurs because fatigue affects the strength and efficiency of respiratory muscles, leading to shallow and rapid breathing, which cannot adequately deliver oxygen to the blood. Fatigue may also cause increased heart rate and impaired blood circulation, reducing oxygen saturation. However, in certain specific work scenarios, the impact of fatigue on SpO_2_ may not be significant. This could be due to the particular nature of the work environment, allowing individuals to maintain relatively stable respiratory and circulatory functions even under fatigue (40, 41). For example, some workers who have undergone specific training or adaptation may be able to maintain higher levels of SpO_2_ during prolonged, high-intensity work. After conducting a one-way ANOVA on multiple data groups, with *P* = 0.001 < 0.05, significant differences in SpO_2_ among different fatigue levels were observed. Specifically, as fatigue increases, there is a significant decrease in SpO_2_ among participants (as shown in Figure 2C). This result underscores the impact of fatigue on SpO_2_, as prolonged work or activity leads to increased oxygen consumption by the body, resulting in a decrease in oxygen content in the blood and subsequently affecting SpO_2_ levels.

#### 3.2.4 VC

VC refers to the air volume between maximal inhalation and maximal exhalation, serving as a crucial indicator for assessing lung function. After prolonged high-intensity exercise or the execution of cognitive tasks, vital capacity decreases significantly (42, 43). The average for adult males is ~3,500 ml (44). Figure 2D depicts that some workers exhibit VC ranging from 5,000 to 6,500 ml, significantly higher than the average. This disparity is attributed to regular physical activity, which strengthens lung muscles and enhances respiratory system function, allowing the lungs to accommodate more air. Consequently, VC often reaches higher levels. After conducting a one-way ANOVA with data from multiple groups, significant differences in VC were observed across different fatigue levels (*P* = 0.000 < 0.05). In extreme fatigue state, VC was generally between 3,000 and 4,000 ml, with some values even lower than the average.

#### 3.2.5 GS

GS refers to the force of hand muscles, reflecting the body's overall strength level and muscle health. The normal range for male grip strength is between 43.5 to 49.5 kg (45). GS tends to decrease after prolonged physical exertion (46). Further analysis of grip strength differences under different levels of fatigue, due to data not meeting homogeneity of variance, Welch's ANOVA was employed, yielding a variance analysis result of *P* = 0.017 < 0.05. This indicates that as fatigue levels increase, the strength of hand muscles is affected, leading to a significant decrease in grip strength among participants (as shown in Figure 2E). Fatigue causes muscle fatigue and depletion of strength, thereby affecting grip strength performance.

#### 3.2.6 RT

RT refers to the time it takes for an organism to respond to a stimulus with a physical action, indicating the temporal gap between receiving the stimulus and reacting to it. The stimulus triggers activity in sensory organs, which is then transmitted through the nervous system to the brain, where it is processed and relayed back to effectors, acting on some external object. It primarily reflects the coordination between the nervous and muscular systems of the human body and their ability to react quickly. Under mild fatigue, reaction times may shorten, but as fatigue worsens, factors such as attentional lapses and decreased inhibitory control may emerge, further contributing to prolonged reaction times (47). A one-way ANOVA yielded a *P*-value of 0.005 <0.05 in the context of different fatigue levels. This indicates that fatigue significantly affects reaction time, with participants exhibiting longer reaction times as fatigue levels increase (Figure 2F).

#### 3.2.7 CFF

CFF refers to the minimum frequency capable of inducing flicker fusion, where the brain perceives continuous light instead of flickering when the speed of light conduction from the eyes to the brain's visual center reaches this frequency. Generally, this frequency ranges between 30 to 55 cycles per second (30). A one-way ANOVA conducted on different fatigue levels yielded *P* = 0.000 < 0.05, indicating a significant impact of fatigue on CFF. Figure 2G illustrates that participants exhibit higher CFF values with increased fatigue. With the accumulation of fatigue, the brain's sensitivity to flicker fusion may decrease, requiring higher frequencies to perceive light flickering.

#### 3.2.8 HR

HR refers to the number of heartbeats per minute in an average person, and the HR in a restful state is known as the resting heart rate. The resting heart rate for a typical adult male typically ranges between 60 to 100 beats per minute (48). Numerous experiments have shown that physical activity can lead to a rapid and temporary increase in HR, shown in Figure 2H; compared to the non-fatigued state, subjects exhibit higher heart rates, generally between 85–90 beats per minute, during extreme fatigue; however, this difference is not statistically significant (*P* = 0.481 > 0.05).

In summary, the differential analysis of eight physiological indicators under different levels of fatigue shows that fatigue significantly affects SpO_2_ (*P* = 0.001), VC (*P* = 0.000), GS (*P* = 0.017), RT (*P* = 0.005), CFF (*P* = 0.000). At the same time, the differences in SBP, DBP, and HR are not significant. Specifically, increasing fatigue levels lead to decreased SpO_2_, reduced VC, decreased Grip, prolonged RT, and increased CFF. However, the variations in SBP, DBP, and HR under different fatigue levels lack statistical significance.

### 3.3 Correlation analysis

**Figure 3:**
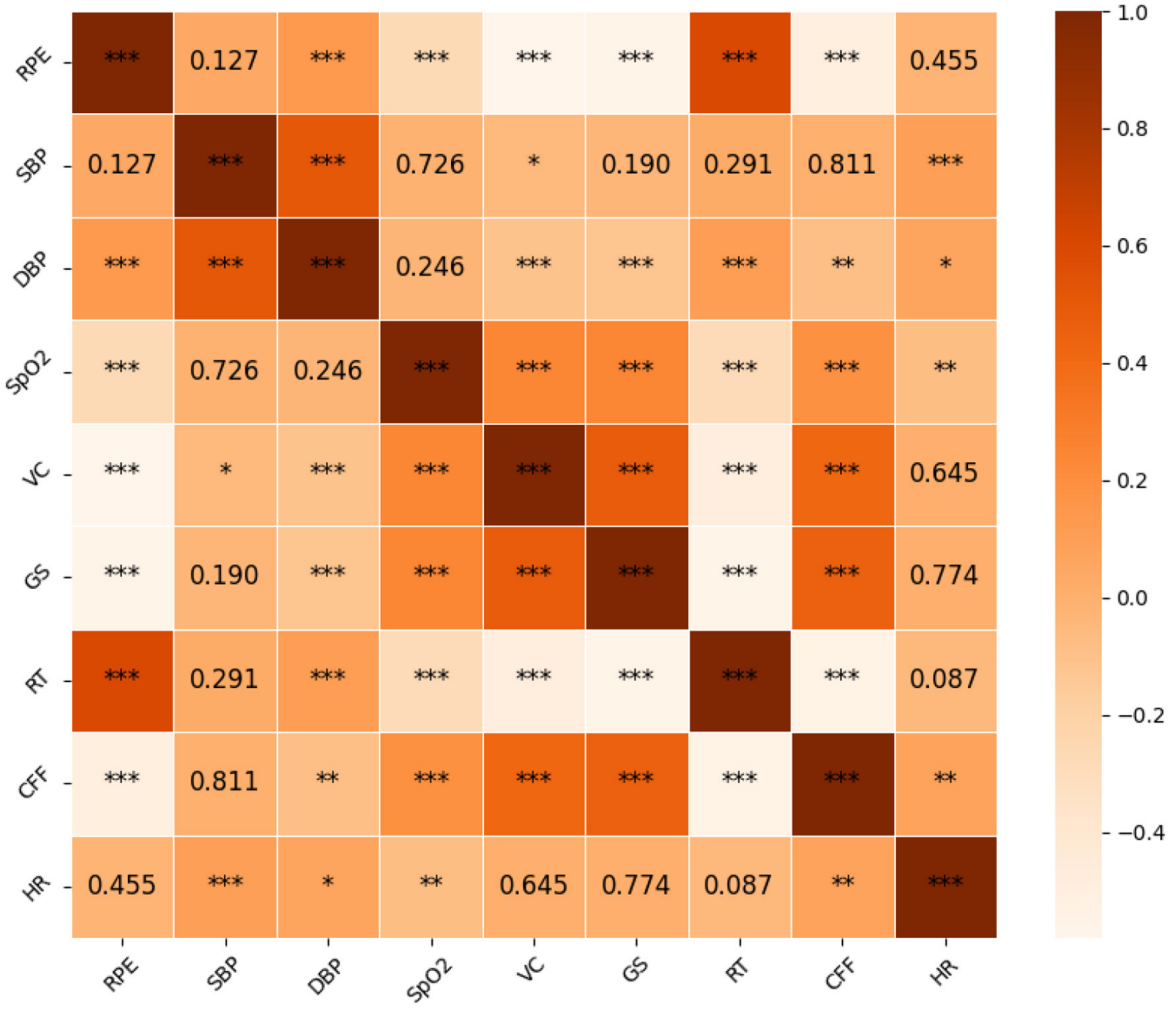
Correlation heatmap. The colors in the chart represent the correlation coefficients, and the numbers represent the *p*-values, *0.01 < *P* < 0.05, **0.001 < *P* < 0.01, and ***P < 0.001.

Spearman correlation coefficients were employed to examine the relationship between physiological indicators and subjective fatigue levels. Findings revealed that fatigue levels exhibited non-significant correlations with HR (*P* = 0.455) and SBP (*P* = 0.127) while demonstrating significant associations with other variables. Subsequent correlation coefficient analyses, depicted in Figure 3, indicated that RT displayed the strongest correlation, followed by VC, which exhibited a closely comparable correlation, both displaying a robust association with fatigue levels. Conversely, DBP and SpO_2_ displayed comparatively weaker correlations with fatigue levels. Positive correlations were observed between fatigue levels and RT and DBP, while negative correlations were noted with VC, CFF, GS, and SpO_2_.

## 4 Fatigue classification

In this study, we used the RPE scale to categorize the participants' fatigue levels into three tiers meticulously: no fatigued state, mild fatigue state, and extreme fatigue state, using the fatigue state as the predictive output for our model. When selecting features, we relied on the correlation analysis results from Section 3.3 and chose six physiological indicators significantly related to fatigue levels as input features for our model. These indicators included DBP, SpO_2_, VC, GS, RT, and CFF. This decision was made because, in the initial evaluation of eight physiological indicators, we found that SBP and HR did not show significant differences across different fatigue levels. Further correlation analysis revealed that SBP and HR lack a clear correlation with the fatigue state, and a fundamental principle of feature selection is to ensure that all selected features are related to the target variable, which in this case is the fatigue state. Therefore, we decided not to include SBP and HR as input features for our model. Although the differences in DBP across various fatigue levels were insignificant, we found a significant correlation between DBP and the fatigue state. This suggests that DBP significantly enhances the predictive power of fatigue state when combined with other physiological indicators. Moreover, including DBP as a feature of the model helps to enhance the model's adaptability and robustness across different individuals and environmental conditions. Even when changes in DBP are not sufficient to serve as a fatigue indicator on their own, they may interact with other features to collectively improve the model's predictive performance (49, 50). To more accurately classify and predict the fatigue states of climbing workers, we employed the XGBoost algorithm to build the fatigue identification model. Data processing and model construction were performed using Python 3.12.

### 4.1 XGBoost algorithm

eXtreme Gradient Boosting (XGBoost) is an enhancement and extension of the gradient boosting tree (GBDT) algorithm. Compared to GBDT, which utilizes only first-order derivatives, XGBoost expands the loss function using the second-order Taylor series, significantly improving the model's prediction effectiveness and operational efficiency. XGBoost algorithm is widely acclaimed in machine learning for its outstanding classification performance and computational efficiency (51, 52). It can automatically discover key factors influencing fatigue states by learning from many sample features and patterns, thereby making accurate classification predictions.

### 4.2 Fatigue identification model based on the XGBoost

**Figure 4:**
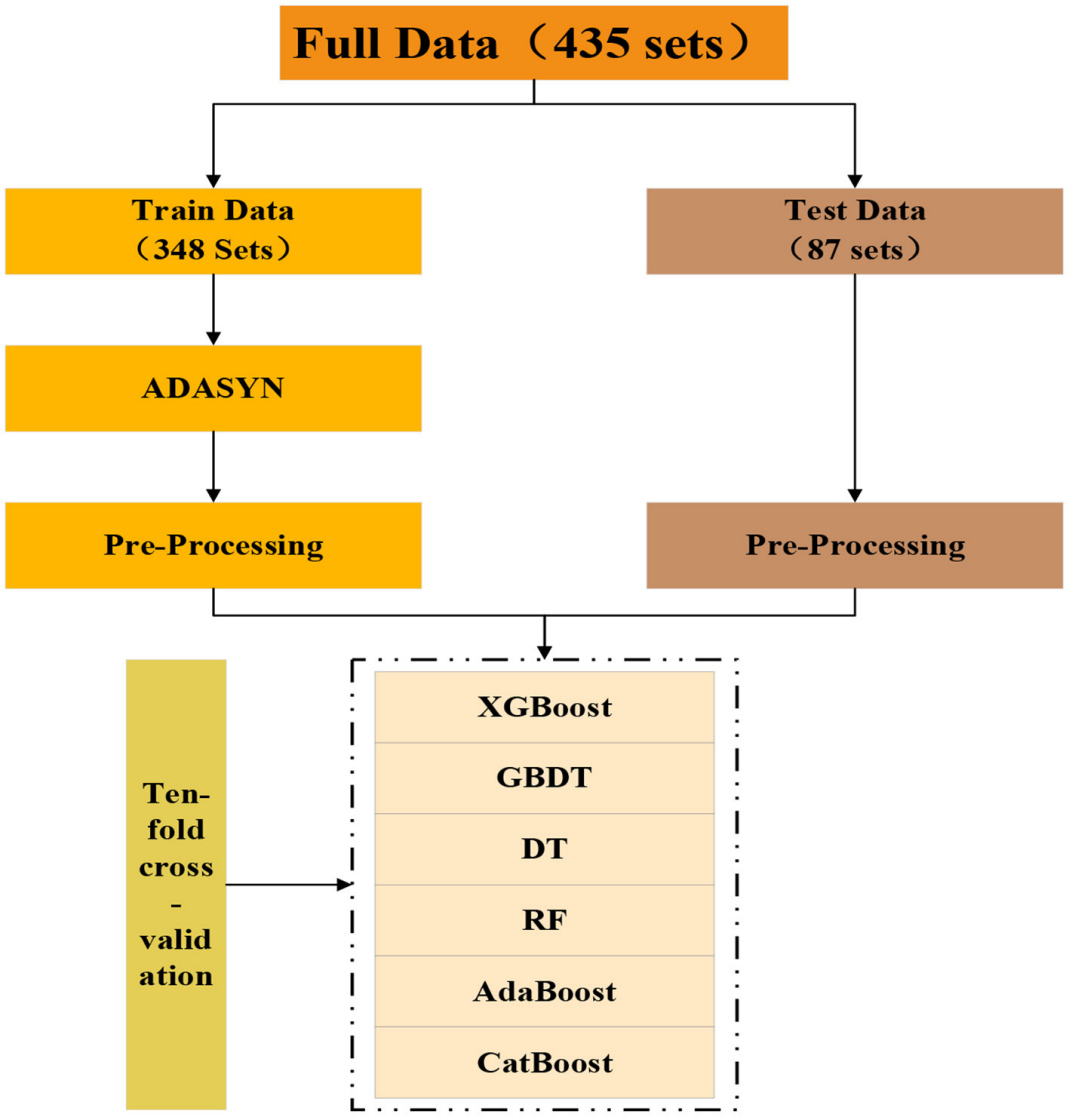
Flow chart of model construction.

The model construction process is depicted in Figure 4. This study randomly divided the data into training and test sets in an 8:2 ratio. Subsequently, the ADASYN algorithm was applied to the training set for sample balancing (53). The original training set comprised 61 samples of no fatigued state, 223 samples of mild fatigue state, and 64 samples of extreme fatigue state. After utilizing the ADASYN algorithm, the training set was expanded to 682 instances, with 228 samples of no fatigued state, 223 samples of mild fatigue state, and 231 samples of extreme fatigue state, significantly enhancing the sample balance of the training set. All features were normalized to mitigate the impact of varying feature scales on the outcomes. Finally, the XGBoost algorithm was employed for model construction, with its base learner being “gbtree,” the optimal model parameters were obtained using a 10-fold grid search cross-validation method, as shown in Table 4. The highest accuracy is 91.95%. It is evident that, out of 87 test datasets, the model for climbing workers accurately identified the fatigue state for 80 datasets.

**Table 4:** Optimal model parameters.

**Parameter name**	**Data range**	**Step length**	**Optimal values of parameters**
learning_rate	[0.05, 0.3]	0.01	0.15
n_estimators	[100, 500]	100	200
max_depth	[1, 10]	1	3
min_child_weight	[1, 10]	1	6
Gamma	[0.05, 0.5]	0.01	0.1

**Figure 5:**
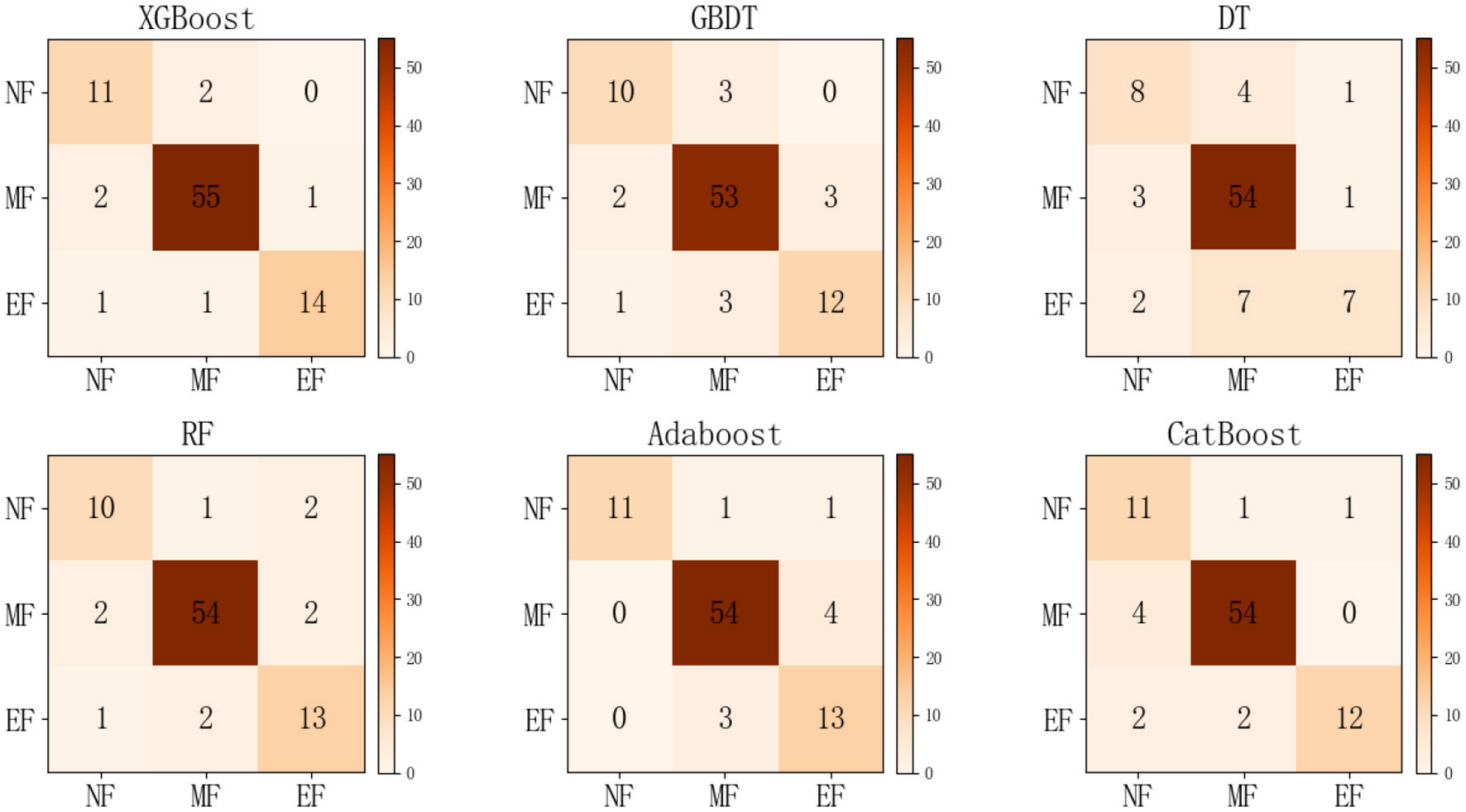
Confusion matrix diagram for different models. XGBoost, eXtreme gradient boosting; GBDT, gradient boosting decision tree; DT, decision tree; RF, random forest; AdaBoost, adaptive boosting; CatBoost, categorical boosting; NF, no fatigue; MF, mild fatigue; EF, extreme fatigue.

To evaluate the performance of the XGBoost model and validate the effectiveness of the proposed method, Gradient Boosting Decision Tree (GBDT), Decision Tree (DT), Random Forest (RF), Adaptive Boosting (AdaBoost), and Categorical Boosting (CatBoost) algorithm models were selected for comparative analysis. The model construction process is similar. Figure 5 presents the confusion matrix of the XGBoost model and the other five classification models. In the confusion matrix depicted in the figure, the diagonal cells correspond to correctly classified observations, while the off-diagonal cells correspond to misclassified observations. Therefore, the XGBoost classification model accurately identifies the fatigue state more accurately than the other five models. Further analysis of the XGBoost Confusion Matrix reveals that 80 state data sets were correctly classified, while seven sets were misclassified. However, the prediction accuracy for no fatigue and extreme fatigue states is low. This lower accuracy may be due to subjective biases in self-reports by participants and the manifestation of symptoms such as irritability, attentional lapses, and sweating during no fatigue and extreme fatigue states. Nevertheless, overall accuracy in fatigue state identification using XGBoost is excellent. This combination of performance indicates that XGBoost achieves appropriate classification results.

Six types of algorithmic models were trained 30 times each (to ensure reproducibility, the random seed for the splitting function was set to 1–30), and the mean values of the prediction accuracy, recall, precision, and F1 score on the test set were taken to evaluate the model performance. TP_i represents the number of samples correctly classified by the model as category (i); FN_i represents the number of samples of the actual category (i) incorrectly classified by the model as other categories; FP_i represents the number of samples of different categories incorrectly classified by the model as category (i). Based on this, the formulas for each evaluation metric are as follows:


(1) $$\text{Accuracy=}\frac{\text{TP}\_\text{1+TP}\_\text{2+TP}\_\text{3}}{\text{total number of samples}}$$

(2) $$\text{Recall}\_\text{i=}\frac{\text{TP}\_\text{i}}{\text{TP}\_\text{i+FN}\_\text{i}}$$

(3) $$\text{Precision}\_\text{i =}\frac{\text{TP}\_\text{i}}{\text{TP}\_\text{i+FP}\_\text{i}}$$

(4) $$\text{F1}\_\text{i =2 }*\;\frac{\text{Precision}\_\text{i }*\text{ Recall}\_\text{i}}{\text{Precision}\_\text{i+Recall}\_\text{i}}.$$

Accuracy is an indicator of the overall performance of the model. However, to avoid the shortcomings of relying solely on accuracy and recall metrics, the F1 score, which combines precision and recall, is adopted to evaluate the model's effectiveness comprehensively. As shown in Table 5, the accuracy of XGBoost reached 89.75%, and the F1 score reached 86.46%, both higher than other models.

**Table 5:** Classification performance of different models.

	**Accuracy**	**Recall**	**Precision**	**F1-score**
XGBoost	0.8982	0.8849	0.8787	0.8646
GBDT	0.7771	0.7971	0.7512	0.7557
DT	0.7543	0.7931	0.7042	0.7232
RF	0.8629	0.8769	0.8431	0.8458
AdaBoost	0.8912	0.8266	0.8531	0.8356
CatBoost	0.8975	0.8849	0.8787	0.8646

## 5 Discussion

We gained deep insights into the physical condition of climbing workers through a detailed descriptive statistical analysis of multiple physiological indicators. The analysis revealed that some workers exhibit elevated SBP and DBP, which may suggest underlying health risks for these individuals. Hypertension is a significant risk factor for cardiovascular diseases, and given the demanding physical and endurance requirements of climbing operations, these high blood pressure values could adversely impact the workers' safety and quality of life. Additionally, we observed significant variation in GS and RT among workers, indicating substantial differences in these indicators among individuals. This variability may stem from fatigue perception and the ability to handle high-intensity work (54, 55). Conversely, SpO_2_ and CFF exhibited lower standard deviations, indicating greater consistency among most participants in these metrics. The stability of these indicators may reflect consistent respiratory and visual system health among workers, with fewer external influences demonstrating marked individual differences (56).

In summary, regular medical examinations are essential to ensure the health and safety of climbing workers. Through more detailed tests, a more accurate assessment of their health status can be made, allowing for targeted interventions to address potential health issues. Furthermore, individual differences should be considered in monitoring and implementing interventions for worker fatigue, and personalized fatigue management plans should be adopted.

The analysis of the RPE scale, subjective fatigue scale, and physiological indicators' variance found that fatigue significantly affects SpO_2_, VC, Grip, RT, and CFF. This is consistent with the conclusion drawn in the literature (57–59). Specifically, with increasing fatigue levels, SpO_2_ exhibited a noticeable decreasing trend. This may be attributed to suppressed functionality across body systems during fatigue, leading to reduced oxygen transport and utilization efficiency in the blood. Additionally, VC was reduced during fatigue, likely due to decreased contraction ability of respiratory muscles, limiting lung expansion and gas exchange efficiency. GS declined noticeably with increasing fatigue, reflecting the gradual weakening of muscle strength and endurance. Moreover, prolonged RT during fatigue directly demonstrates its impact on the nervous system. Lastly, the increase in CFF reveals changes in the visual system during fatigue, potentially impairing visual perception and judgment among workers. These findings deepen our understanding of how fatigue affects the physical condition of climbing workers and provide a vital scientific basis for constructing a fatigue recognition model for these workers.

Notably, although fatigue significantly impacts multiple physiological indicators, variance analysis results indicate that fatigue does not considerably affect SBP, DBP, and HR. This may be because numerous factors influence changes in these indicators. Therefore, when assessing the physical condition of workers, a comprehensive consideration of multiple indicators' changes is necessary to obtain a more thorough understanding.

In conclusion, employing a combined objective and subjective approach allows for an accurate assessment of the fatigue states of climbing workers. Based on experimental results, we further developed an XGBoost predictive recognition model. This model, trained and optimized with substantial data, can efficiently and accurately identify the current fatigue states of workers. The model can swiftly analyze and provide fatigue state determinations by inputting workers' physiological indicator data.

## 6 Conclusions

This study quantifies the fatigue levels of power pole climbers by collecting subjective fatigue scales and various physiological data on-site. An XGBoost algorithm was also employed to establish a fatigue recognition model for climbing operations. The study found that fatigue significantly affects SpO_2_, VC, Grip, RT, and CFF, while its impact on SBP, DBP, and HR is insignificant. Compared to models like GBDT, the XGBoost identification model performed the best, with a classification accuracy of 89.75%.

The study has some limitations that need to be addressed in future research. First, the number of subjects is relatively small. To fully leverage the advantages of machine learning, a large-scale dataset is required, which can be obtained in subsequent applications of the test methods used in this study. Second, the study mainly collected physiological indicators such as SBP, DBP, and SpO_2_, which are widely used in the medical and physiological fields, making their acquisition relatively straightforward. However, various factors may influence these indicators, leading to changes that may not fully reflect the fatigue state. Therefore, future research should consider incorporating more objective physiological and psychological measurement indicators to comprehensively assess participants' fatigue states (60, 61). Third, the results of the RPE scale heavily depend on participants' subjective perceptions. Acknowledging fatigue may be perceived as a weakness or deficiency, this motivation affects their judgment of fatigue. This indicates that human motivation should be noticed when assessing fatigue. Fourth, the number of participants at different fatigue levels is unequal. Future efforts could involve broader recruitment and selection to obtain more participants with different fatigue levels.

In theoretical terms, this study differs from past analyses of worker fatigue, which often focused on laboratory environments or simulated scenarios. While these studies provide some theoretical basis, they must improve in reflecting the complexity and diversity of actual work environments. In contrast, this study, based on actual work scenarios, particularly in the high-intensity, high-risk field of climbing operations, delved into the fatigue states of workers in actual work processes. Additionally, the study emphasizes the feasibility of identifying individual fatigue based on physiological signals. Traditional fatigue assessment methods often rely on subjective questionnaires, which, although simple and practical, need more subjectivity and accuracy. In contrast, fatigue recognition methods based on physiological signals can objectively reflect workers' physiological states, thereby providing more accurate and reliable fatigue assessment results.

In practice, this study can provide a basis for personalized fatigue management for power pole climbers. By employing fatigue recognition models, managers can more reasonably schedule rest time for workers, devise personalized rest plans, and ensure timely rest for workers when fatigue reaches a certain level, thus avoiding the adverse effects of excessive fatigue on health. Climbing operations inherently involve certain risks, and if workers are tired, their reaction speed and judgment abilities may decline, thereby increasing the risk of accidents. Using this model, we can promptly detect workers' fatigue states and take corresponding measures to minimize the likelihood of accidents occurring to the greatest extent possible.

## Data Availability

The raw data supporting the conclusions of this article will be made available by the authors, without undue reservation.
